# Mathematical model for frequency modulation in the respiratory network

**DOI:** 10.1186/1471-2202-12-S1-P25

**Published:** 2011-07-18

**Authors:** Natalia Toporikova, Robert Butera

**Affiliations:** 1 Laboratory for Neuroengineering, Georgia Institute of Technology, Atlanta, GA, 30332-0250, USA; 2 School of Electrical and Computer Engineering, Georgia Institute of Technology, Atlanta, GA, 30332-0250, USA

## 

Neuromodulators, such as amines and neuropeptides, alter the activity of neurons and neuronal networks. In this work, we investigate how neuromodulators which activate G-proteins and second messenger systems can modulate the frequency of bursting neurons in a critical portion of the respiratory neural network, the pre-Bötzinger complex (pBC). Inspiratory neurons in the pBC produce a regular bursting rhythm in phase with the activity of inspiratory muscles in the diaphragm. These neurons are a vital part of the ponto-medullary neuronal network, which generates a stable respiratory rhythm [1]. The frequency of pBC depends on the concentration of Serotonin (5-HT) and Substance P (SP), neurotransmitters released by the nearby Raphe nucleus. Both neurotransmitters, 5-HT and SP, affect pBC neurons by activating receptors coupled with the *G_q_* protein pathway, thereby inducing *Ca^2^*^+^ release from the Endoplasmic Reticulum (*ER*).

We have previously developed a mathematical model of the pBC neuron, which incorporates explicit activation of *G_q_*-protein coupled receptors, and have shown that activation of these receptors can result in *Ca^2^*^+^ oscillations in the dendritic compartment [2]. The model exhibits two independent bursting mechanisms – bursting in the soma depends on persistent sodium current, whereas bursting in the dendrite follows *Ca^2^*^+^ oscillations. It has been recently found that the connection between the pBC and the Raphe nucleus is bi-directional: not only does the Raphe nucleus release 5-HT and SP to modulate the frequency of pBC neurons, but also the rhythmic activity in the pBC increases the firing of Raphe neurons [3]. In this work, we extend our model to a network of pBC neurons while incorporating this newly discovered interaction between Raphe and pBC nuclei.

**Figure 1:**
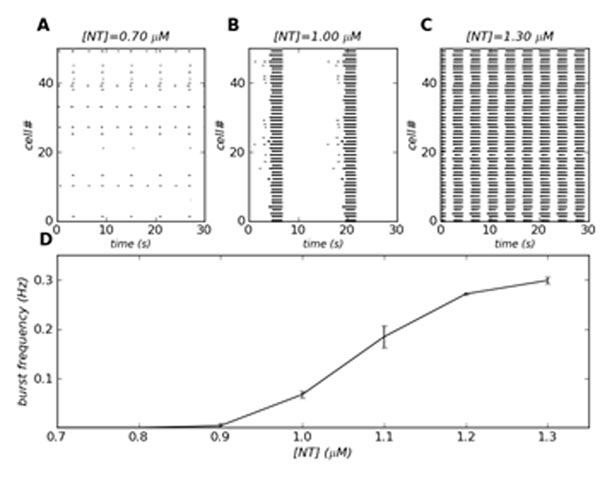
Inspiratory frequency modulation of pBC by excitatory neurotransmitters, which act on *G_q_*-coupled receptor. *[NT]* represents neurotransmitter concentration. (A-C) Example of raster plots for three different neurotransmitter concentrations. (A) Rhythmic activity is absent for low concentration of neurotransmitter (*[NT]*=*0.7 µM*). (B) Increase in neuromodulatory tone (*[NT]*=*1 µM*) results in a slow bursting rhythm. (C) Elevation of neurotransmitter concentration (*[NT]*=*1.3µM*) increases the burst frequency in pBC. (D) Burst frequency of pBC neurons as a function of neurotransmitter concentration. The vertical bars represent the standard error from an average of 10 network simulations.

Using a simulated 50-cell network of excitatory connected pBC neurons with a heterogeneous distribution of persistent sodium conductance and ER Ca^2+^, we show that a tonic release of neurotransmitters acting on the *G_q_* protein pathway increases the number of intrinsic bursters in such a network. However, when we simulated the application of different concentrations of SP or 5-HT, there was no dose-dependent frequency modulation. We then added a positive feedback between the Raphe excitability and pBC activity, representing the release of neurotransmitters from Raphe, and found that this feedback induces frequency modulation the pBC neurons (Figure 1). Thus, our model shows that the frequency of the respiratory rhythm can be modulated via phasic release of 5-HT and SP from the Raphe nucleus.
